# Supplementary material for: Identification of photoperiod-induced specific miRNAs in the adrenal glands of Sunite sheep (Ovis aries)
Source: Front Vet Sci. 2022 Jul 22;9:888207. doi: 10.3389/fvets.2022.888207 (PMC9354845; doi:10.3389/fvets.2022.888207)
Supplement: Supplementary file 1 [file Data_Sheet_1.ZIP › Supplementary file/miRDeep2 file.zip!/miRDeep2 file/Know_Structure/SPLP42A2/oar-mir-30d.pdf]

miRBase precursor : oar-mir-30d
Total read count : 232702
oar-miR-30d read count : 231788
remaining reads : 914

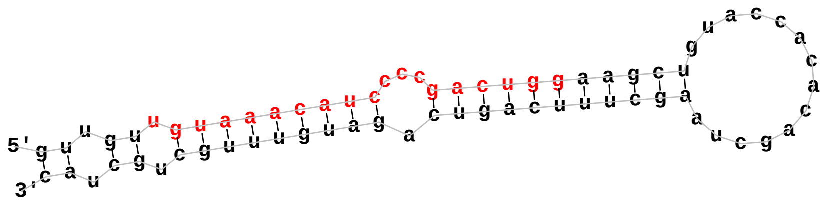

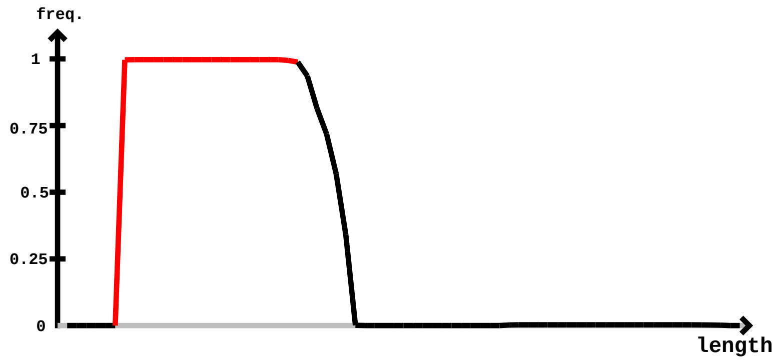

oar-miR-30d

```
5'- guuguuguaaacauccccgacuggaagcuguaccacacagcuaagcuuucagucagauguuugcugcuac   -3'    exp
    ...((.(((((((((...(((((((((((..............))))))))))).))))))))).))...    reads      mm         sample
    ....uuguaaacauccccgacugga.............................................    1          0          seq
    ....uuguaaacauccccgacuggaagcu.........................................    2          0          seq
    .....uguaaacaucccUgacu................................................    5          1          seq
    .....uguaaacaAccccgacu................................................    1          1          seq
    .....uguaaacauccccgacu................................................    649        0          seq
    .....uguaaacauccccgGcu................................................    2          1          seq
    .....uguaaacaucUccgacu................................................    2          1          seq
    .....uguaaacauccccgacug...............................................    1429       0          seq
    .....uguaaacauccccgacugg..............................................    12222      0          seq
    .....uguaaacauccccgacugga.............................................    28100      0          seq
    .....uguaaacauccccgacuggaa............................................    22483      0          seq
    .....uguaaacauccccgacuggaag...........................................    34806      0          seq
    .....uguaaacauccccgacuggaagc..........................................    53124      0          seq
    .....uguaaacauccccgacuggaagcu.........................................    78813      0          seq
    .....uguaaacauccccgacuggaagcug........................................    200        0          seq
    .....uguaaacauccccgacuggaagcugu.......................................    30         0          seq
    .....uguaaacauccccgacuggaagcugua......................................    2          0          seq
    .....uguaaacauccccgacuggaagcuguaccaca.................................    1          0          seq
    ......guaaacauccccgacugg..............................................    4          0          seq
    ......guaaacauccccgacugga.............................................    13         0          seq
    ......guaaacauccccgacuggaa............................................    8          0          seq
    ......guaaacauccccgacuggaag...........................................    16         0          seq
    ......guaaacauccccgacuggaagc..........................................    38         0          seq
    ......guaaacauccccgacuggaagcu.........................................    59         0          seq
    .......uaaacauccccgacuggaa............................................    1          0          seq
    .......uaaacauccccgacuggaagcu.........................................    7          0          seq
    ........aaacauccccgacuggaa............................................    1          0          seq
    ........aaacauccccgacuggaagcu.........................................    2          0          seq
    .............................................cuuucagucagauguuugc......    20         0          seq
    .............................................cuuucagucagauguuugcu.....    81         0          seq
    .............................................cuuucagucagauguuugcug....    70         0          seq
    .............................................cuuucagucagauguuugcugc...    110        0          seq
    .............................................cuuucagucagauguuugcugcu..    217        0          seq
    .............................................cuuucagucagauguuugcugcua.    3          0          seq
    ..............................................uuucagucagauguuugc......    1          0          seq
```

oar-miR-30d

```
guuguuguaaacauccccgacuggaagcuguaccacacagcuaagcuuucagucagauguuugcugcuac
..............................................uuucagucagauguuugcu.....    8          0          seq
..............................................uuucagucagauguuugcug....    8          0          seq
..............................................uuucagucagauguuugcugc...    35         0          seq
..............................................uuucagucagauguuugcugcu..    125        0          seq
..............................................uuucagucagauguuugcugcua.    1          0          seq
...............................................uucagucagauguuugcugc...    2          0          seq
```
